# Current Status and Challenges of Diaphragm Pacing in Japan: A Systematic Review of Case Reports

**DOI:** 10.7759/cureus.80776

**Published:** 2025-03-18

**Authors:** Ryoko Yamauchi, Ryuichi Ohta, Chiaki Sano

**Affiliations:** 1 Rehabilitation, Kanagawa Rehabilitation Hospital, Atsugi, JPN; 2 Community Care, Unnan City Hospital, Unnan, JPN; 3 Community Medicine Management, Shimane University Faculty of Medicine, Izumo, JPN

**Keywords:** congenital central hypoventilation syndrome, diaphragm pacing, japan, quality of life, respiratory insufficiency, spinal cord injuries, ventilator weaning

## Abstract

Diaphragm pacing (DP) is a therapeutic intervention for ventilator-dependent patients with spinal cord injury (SCI) and congenital central hypoventilation syndrome (CCHS). Despite its availability, clinical adoption in Japan remains limited. This systematic review assesses the current state of DP in Japan, its outcomes, and its associated challenges by analyzing 10 case reports from the Ichushi database. Patients ranged from four to 50 years old, predominantly male (90%), with cervical SCI primarily due to traffic trauma (63%). DP was introduced with a median of 24 months post-injury. All patients achieved partial or complete ventilator weaning, with 27% achieving full independence. Reported benefits include improved quality of life (QOL), mobility, and social reintegration. However, complications such as respiratory muscle fatigue (54%), ventilatory issues in a seated position (18%), and pain due to stimulation (9%) were observed. Barriers to DP implementation in Japan include delayed introduction, limited interdisciplinary collaboration, and inadequate home care support. Early DP initiation, structured follow-up, and telemedicine integration could enhance outcomes. Further research is needed to establish standardized guidelines and optimize DP use.

## Introduction and background

Diaphragm pacing (DP) is a therapeutic intervention used to address apnea in conditions such as spinal cord injury (SCI) and congenital central hypoventilation syndrome (CCHS) [1]. In Japan, DP received insurance coverage in 2019 and has been implemented clinically since 2020 [2]. The NeuRx Diaphragm Pacing System by Synapse Biomedical (Oberlin, Ohio, United States), the DP device currently approved for insurance coverage, has been utilized in the United States since 2002, with over 2,000 documented cases [3].

**Figure 1:**
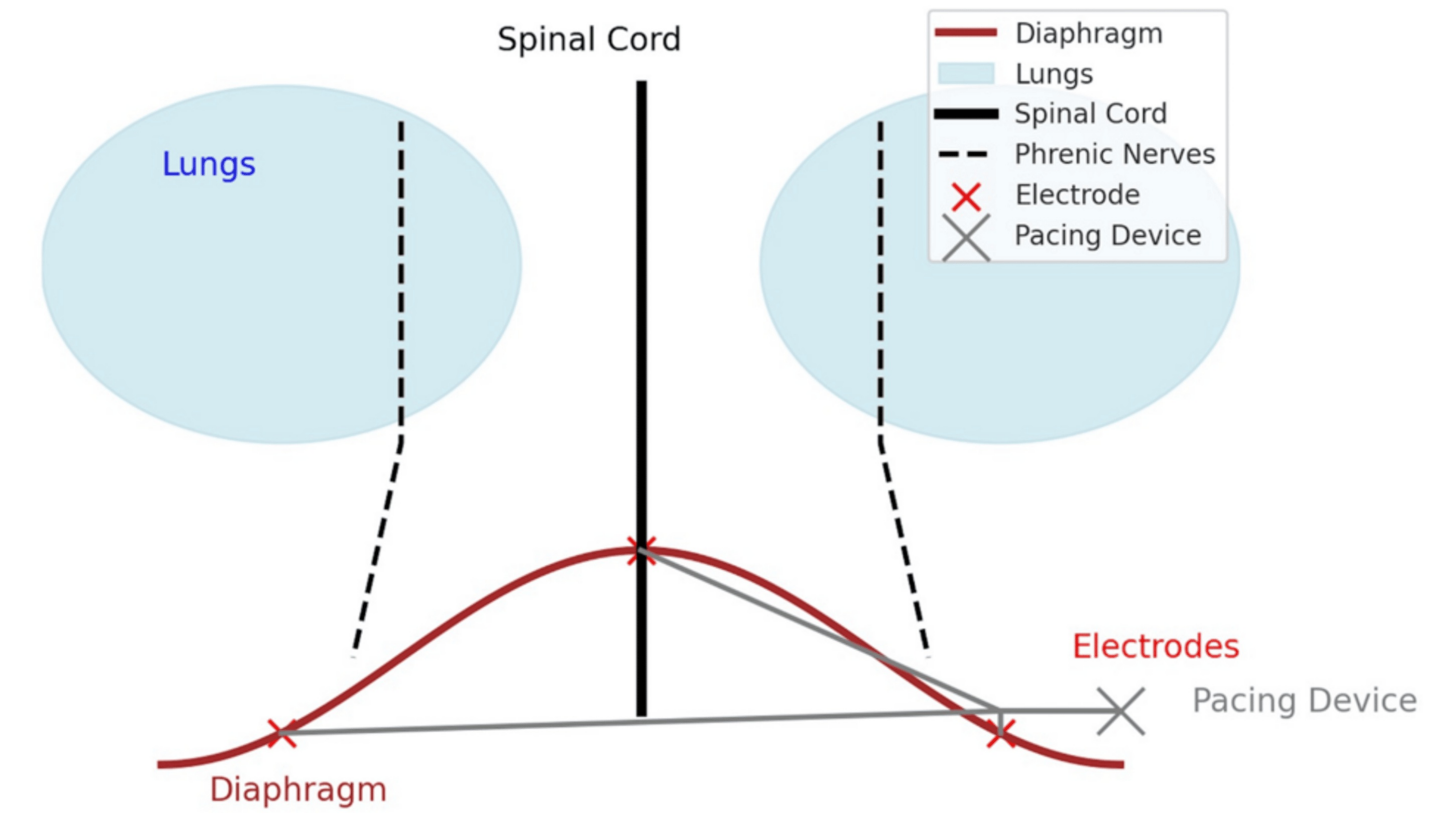
Diaphragm pacing mechanism This figure illustrates the diaphragm pacing mechanism, a technique used to stimulate the diaphragm in patients with ventilatory insufficiency. The diaphragm (brown curved structure) is innervated by the phrenic nerves (dashed black lines), which originate from the cervical spinal cord and attach to the diaphragm. Electrodes (red dots) are surgically implanted directly onto the diaphragm and connected via insulated leads to an external pacing device (gray), located to the right of the spinal cord. The pacing device delivers controlled electrical impulses to the electrodes, stimulating the diaphragm and inducing rhythmic contractions to facilitate breathing. The lungs (light blue) expand due to diaphragm activation, assisting with respiration. Figure Credit: Ryuichi Ohta

Figure 1 shows the mechanism of DP.

Historically, the Avery Diaphragm Pacing System (Avery Laboratories, Inc., Commack, New York, United States) was implanted in Japan as early as the 1980s [4]. However, several barriers hindered its widespread use [4]. These included the lack of insurance coverage, high out-of-pocket costs, unavailability of manufacturing and distribution within Japan, and inadequate post-implantation support systems [5]. Consequently, DP adoption remained limited during that period.

In Japan, the NeuRx Diaphragm Pacing System obtained regulatory approval for SCI and CCHS in 2017, followed by its inclusion in the national insurance system in 2018 [3]. Clinical implementation began in 2020. DP offers the potential for ventilator independence by enabling negative-pressure breathing similar to spontaneous respiration [3]. This method has benefits such as reduced airway secretions and prevention of respiratory-related infections, including ventilator-associated pneumonia [6].

In July 2023, the Japanese Society for Spine Injury Medicine developed comprehensive guidelines for adequately using DP systems [2]. These guidelines include recommendations on indications, procedural steps, and facility and physician qualifications, advancing readiness for clinical utilization. However, limited clinical experience with DP in Japan's healthcare settings has hindered its widespread adoption.

Given its potential benefits and the underutilization of DP, there is a pressing need to accumulate medical insights and evidence regarding its use. In this study, we conduct a systematic review to evaluate the current state of DP use in Japan, its outcomes, and its associated challenges. This investigation aims to provide an academic perspective on the role and potential of DP in Japan's healthcare system.

## Review

Method

The systematic review was performed using Ichushi, a Japanese academic search engine. Search terms included "diaphragm pacing" and "original articles". The inclusion and exclusion criteria used for the review are detailed in Table 1.

**Table 1:** Inclusion and exclusion criteria SCI: spinal cord injury; CCHS: congenital central hypoventilation syndrome; DP: diaphragm pacing

Criteria	Inclusion	Exclusion
Population	SCI or CCHS patients	Other patients and animals
Intervention	Clinical application of DP	The other interventions
Outcome	Post-implantation rehabilitation outcomes	Other outcomes
Type of study	Case reports	Non-empirical studies (editorials, news)

The primary investigator (RY) extracted data from each original article. Next, investigators (RO and CS) checked the extracted data. Extracted data were categorized into age, gender, injury site, cause of injury, time to DP implementation, care setting, and outcome.

Analysis

Quantitative and qualitative data are presented as descriptive statistics.

Result

Search Strategy

**Figure 2:**
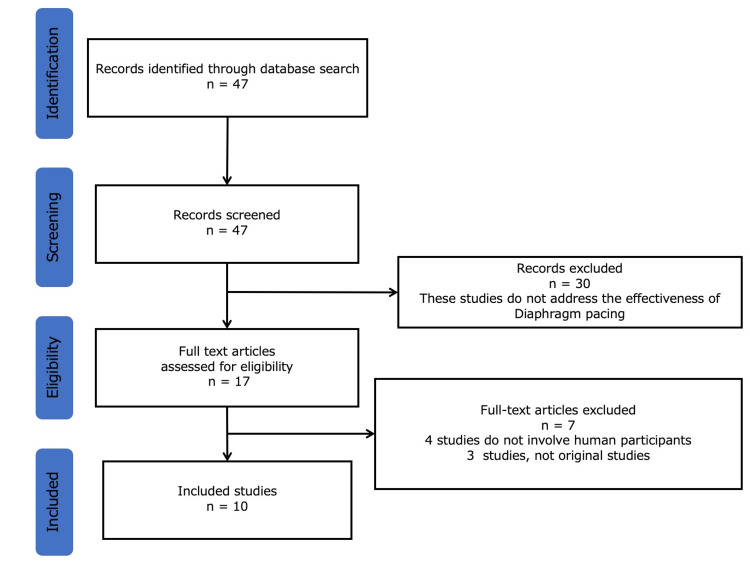
The selection flow DP: diaphragm pacing

We conducted a search using the online database Ichushi, using the keywords "diaphragm pacing" and "case report". The selection flow is shown in Figure 2.

Forty-seven articles were identified. We screened these articles based on the inclusion and exclusion criteria, ultimately including 10 in our study results (Table 2).

**Table 2:** The included articles with various categories C: cervical spine; SCI: spinal cord injury; CCHS: congenital central hypoventilation syndrome; DP: diaphragm pacing; TV: tidal volume

First author (publication year)	Age	Gender	Injury site	Cause of injury	Time to DP implementation	Care setting	Outcome
Kawai et al. (1987) [7]	4	Male	C1	Traffic trauma	6 months	Hospital	DP use extended to 4 hours, enabling wheelchair mobility both inside and outside the hospital
Sakai et al. (1989) [8]	39	Male	C4/5	Fall	31 months	Not stated	DP use extended to 4 hours. Combined ventilator use. Enabled wheelchair mobility
Narita et al. (1991) [9]	22	Male	Upper cervical spinal cord	Traffic trauma	16 months	Home	Gradual improvement in quadriplegia. Used DP at night for central sleep apnea syndrome. Fully weaned off ventilator
Koike (1992) [10]	24	Female	C4	Traffic trauma	24 months	Not stated	Continuous DP use extended to 1 hour over 6 months. Enabled walks in a wheelchair, expanding the living environment
Kurashima (1992) [11]	47	Male	C2	Traffic trauma	111 months	Home	DP use extended to 12 hours daily. Ventilator use continued. Enabled outings by car and weekly outpatient visits for bathing
Takei et al. (1992) [12]	35	Male	C2	Fall	36 months	Not stated	DP use exceeded 10 hours. Combined ventilator use at night due to patient anxiety. Promoted active mobilization
Maekawa (1993) [13]	22	Male	C3	Traffic trauma	29 months	Home	Quadriplegia recovery enabled independent walking. Initially used DP for sleep apnea syndrome; later discontinued DP with full recovery and tracheostomy closure
Niino et al. (1995) [14]	40	Male	C1	Atlantoaxial dislocation with basal skull depression	13 months	Hospital	Continuous DP use extended to 9 hours. Combined with ventilator. Enabled sitting for TV viewing and wheelchair mobility
Nagai et al. (1996) [15]	50s	Male	C2	Traffic trauma	108 months	Home	Ventilator use limited to nighttime due to lack of alarm function. Able to travel by car and attend outpatient visits. Home renovation and weekly visiting nursing introduced
Nagai et al. (1996) [15]	39	Male	C2	Fall	24 months	Facility	Combined use of ventilator and DP enabled outpatient visits. Entered a care facility due to family circumstances
Morita et al. (2007) [16]	20	Male	C2	Traffic trauma	18 months	Home	Tracheostomy closure achieved, completely weaned off ventilator, and wheelchair mobility improved

Patient Characteristics

The patients ranged in age from four years to their 50s, with a median age of 35. Most cases were male (10 cases, 90%). The predominant cause of SCI was traffic accidents (seven cases, 63%), followed by falls (three cases, 27%) and disease (one case, 9%). Injuries were predominantly located in the cervical spine (C1-C4), with C2 being the most common site (five cases, 45%). The time from injury to DP implementation ranged from six to 108 months, with a median of approximately 24 months.

DP Implementation and Outcomes

DP implementation was gradually extended in all cases while monitoring conditions such as dyspnea, pain, and respiratory muscle fatigue. Follow-up data indicated the effective use of DP for at least six months, ranging from a minimum of one hour to complete ventilator weaning [9,10,13,16]. In cases capable of complete weaning, nighttime DP use was continued due to concerns such as lack of alarm function or patient anxiety [12,15]. Partial or complete ventilator independence was achieved in all 11 cases. Complete independence was achieved in three cases (27%), especially in early implementation cases (within 1-2.5 years post-injury). Two of these cases also showed improvement in quadriplegia [9,13].

Complications and Countermeasures

Reported complications included decreased ventilation in a seated position (two cases, 18%), pain due to DP stimulation (one case, 9%), lead replacement due to surgical site infection (one case, 9%), and dyspnea or fatigue attributed to respiratory muscle fatigue (six cases, 54%). Ventilation issues in a seated position were resolved using abdominal binders [10,14]. For fatigue, temporary discontinuation of DP allowed muscle recovery. Psychosocial concerns regarding ventilator independence were addressed through adequate explanation, pre-DP training, and psychological support. Obstructive sleep apnea during DP use was managed with lifestyle modifications, rehabilitation, vitamin B supplementation, and avoidance of alcohol and sedatives [13].

Prognosis

All cases reported improved quality of life (QOL) and activities of daily living (ADLs), including enhanced mobility and social reintegration. Most patients (five cases, 45%) were managed at home, while others received care in hospitals (two cases, 18%) or facilities (one case, 9%). Details of specific medical services and community support were generally lacking, with home care services mentioned only in two instances [11,15].

Discussion 

In this study, we conducted a systematic review to investigate the current status and challenges of DP in Japan. The findings suggest that DP has the potential to facilitate ventilator weaning and improve QOL. On the other hand, challenges such as insufficient coordination among medical institutions, interdisciplinary collaboration, and a lack of home care support were highlighted.

After DP implantation, it is necessary to gradually extend the usage time while assessing patient anxiety related to ventilator weaning and respiratory muscle fatigue to achieve and maintain the desired ventilation state [5,17]. DP requires collaboration among specialists treating underlying diseases such as SCI and CCHS [6,18]. This includes spine surgeons, neurosurgeons, pediatricians, neonatologists, emergency physicians, gastrointestinal or pediatric surgeons for laparoscopic implantation, pulmonologists, and rehabilitation physicians for postoperative management [19]. This systematic review indicated that DP introduction and conditioning were performed at the same facility [8,10,13]. This setup may allow timely conditioning adjustments during complications such as pain, discomfort from stimulation, or reduced ventilation during mobilization. 

From the perspective of Japan's healthcare framework, delineating roles among healthcare institutions is essential. In the Japanese cases, DP implantations were performed at an acute care hospital equipped to handle emergencies and included the necessary specialties [7,11]. Subsequent conditioning and integration into daily life were conducted at a chronic care hospital [7,11]. This systematic review demonstrates that seamless care transitions between institutions aligned with their respective roles are desirable. Effective communication and collaboration between the university hospital performing DP and the rehabilitation hospital managing post-implantation care are pivotal [18,20,21]. As this systematic review shows, multiple meetings before surgery, with evaluations of respiratory and physical functions, enabled all team members to share the risks and benefits of DP [16]. Establishing a follow-up system for DP management, potentially through telemedicine, has been suggested to enhance outcomes, given the limited number of facilities offering DP in Japan [22]. Building a continuous collaboration framework utilizing videoconferencing or online consultations remains a key challenge for safely expanding DP use.

DP techniques include intrathoracic diaphragm pacing (IT-DP) and intraperitoneal diaphragm pacing (IP-DP). Both methods show high ventilator weaning rates of approximately 72-96%, consistent evidence of improved QOL by restoring more physiological respiration [4]. By replicating negative-pressure breathing, DP can potentially reduce ventilator-associated pneumonia risk and restore physiological respiratory states [4]. However, the results of this review indicated that respiratory muscle fatigue and reduced ventilation during positional changes were identified as challenges [7,14]. Respiratory muscle fatigue is likely due to diaphragmatic atrophy, as prolonged ventilator use can lead to functional deterioration and disuse atrophy of the diaphragm, increasing ventilator dependency with longer usage durations [5,6,17]. Early introduction of DP could prevent diaphragmatic atrophy and facilitate earlier ventilator weaning. Studies have shown a higher proportion of complete ventilator weaning following DP introduction [23].

This systematic review revealed that DP introduction took approximately two years post-injury, primarily due to barriers such as high costs (before insurance coverage), the unavailability of devices in Japan, and the lack of postoperative support systems [12]. In the current cases, DP was introduced 15 months post-injury [12]. Gradual extension of DP usage time led to complete ventilator weaning in less than a month, significantly earlier than in previous literature [7,8,12]. Early DP introduction before significant diaphragmatic atrophy and the patient's robust baseline capacity likely contributed to this outcome [3]. Early DP introduction in traumatic cervical SCI cases may reduce ventilator dependence and improve outcomes [19]. DP also promotes neuromuscular plasticity, potentially leading to enhanced spontaneous diaphragmatic activation and respiratory function, underscoring the need for developing new respiratory rehabilitation models using DP [18]. Among acute SCI patients, 67% experience respiratory complications, with incidences as high as 84% in cervical levels C1-C4 and 60% in C5-C8 injuries [24]. Early DP introduction in the acute phase could improve long-term prognoses and QOL for these patients.

Ventilation reduction during positional changes is another challenge. This review suggested that increasing DP output or using abdominal binders could address this issue. Reduced tidal volume in the seated position is attributed to visceral downward displacement, reducing expiratory assistance [25]. Adjusting pacing output requires specialized equipment and may cause pain, highlighting the need for flexible settings and tailored interventions [25]. In addition, psychological support is also crucial for DP patients, as differences in breathing patterns compared to ventilator-assisted respiration and anxiety over ventilator weaning can manifest as dyspnea or hyperventilation symptoms [26]. Psychological distress may affect DP usage despite normal ventilation function, underscoring the need for appropriate psychological support [27].

This study has several limitations. First, the number of cases examined is small. This systematic review included a limited number of cases, which may not comprehensively reflect the nationwide implementation status of DP in Japan. Additionally, information on the efficacy and challenges of DP was primarily based on case reports, lacking standardized evaluation criteria, which limits the generalizability of the findings. Second, there is a paucity of detailed data on home care and regional healthcare systems. Further investigation is needed to identify specific challenges and success factors in home care and visiting nurse support. Third, the systematic review relied on literature primarily sourced from Japan, potentially lacking an international perspective. Future studies should focus on comprehensive data collection and analysis involving more cases to establish robust evidence on the efficacy and challenges of DP. Comparative analyses with international literature and long-term outcome assessments are essential for establishing guidelines for DP implementation in Japan.

## Conclusions

DP therapy remains underutilized in Japan, with limited case reporting and minimal data on post-discharge home care. This study underscores the importance of post-implantation adjustments and respiratory management for successful DP integration. As adoption increases, ensuring safe home care and educating families and regional healthcare providers will be essential to improving patient outcomes.
